# Cholesterol intake and serum total cholesterol levels are not associated with total testosterone levels in men: a cross-sectional study from NHANES 2013–2014

**DOI:** 10.1186/s12944-023-01928-7

**Published:** 2023-10-05

**Authors:** Gederson K. Gomes, Flávia M. S. de Branco, Heitor O. Santos, Jaqueline L. Pereira, Fábio L. Orsatti, Erick P. de Oliveira

**Affiliations:** 1 Laboratory of Nutrition, Exercise and Health (LaNES), School of Medicine, Federal University of Uberlandia (UFU), Uberlandia, Minas Gerais 38400902 Brazil; 2 Department of Nutrition, School of Public Health, University of São Paulo (USP), São Paulo - SP, Brazil; 3 Exercise Biology Research Group (BioEx), Federal University of Triangulo Mineiro (UFTM), Uberaba, MG Brazil; 4 Department of Sport Sciences, Federal University of Triangulo Mineiro, Uberaba, MG Brazil; 5 School of Medicine, Federal University of Uberlandia (UFU), Uberlandia, Minas Gerais Brazil

**Keywords:** Androgens, Cholesterol, Hypogonadism, Lipids, Sex hormones, Testosterone

## Abstract

**Background:**

Testosterone (T) is an anabolic hormone crucial to the structure and function of skeletal muscle. Testosterone is partially synthesized from cholesterol, but little is known about the relationship of cholesterol intake and serum cholesterol with T levels.

**Aim:**

To investigate whether cholesterol intake and serum total cholesterol (TC) levels are associated with serum total testosterone (TT) levels in men.

**Methods:**

A cross-sectional study enrolling 1996 men aged 20 to 80 years from National Health and Nutrition Examination Survey (NHANES) 2013–2014 was carried out. Diet assessment was performed using two 24-h food recalls, and TT levels were measured by liquid chromatography coupled with tandem mass spectrometry. Regression analyses were performed to evaluate whether TT was associated with cholesterol intake and serum TC levels.

**Results:**

Neither cholesterol intake nor serum TC levels were associated with TT levels in unadjusted and adjusted analyses (adjustment for energy, total fat and alcohol intake, smoking, age, physical activity, family income, marital status, race, educational level, diabetes, hypertension, and body mass index).

**Conclusion:**

Dietary cholesterol intake and TC levels are not associated with TT levels in men from the USA.

**Supplementary Information:**

The online version contains supplementary material available at 10.1186/s12944-023-01928-7.

## Introduction

Testosterone (T) is an important sex hormone for reproductive factors, acting in the process of male genital development and secondary sexual characteristics [1, 2]. T is also fundamental to skeletal muscle structure and function [3]. In males, T production increases rapidly under stimulation of anterior pituitary gonadotropic hormones at the onset of puberty, but values tend to decline after 50 years and may decrease by 20% to 50% at 80 years [4].

Low levels of total testosterone (TT) are associated with increased body fat and decreased skeletal muscle mass during aging [5, 6], as well as an increased likelihood of having low libido and erectile dysfunction [7]. In addition, recent studies show that low TT levels are associated with underlying comorbidities and potentially higher mortality risk in men [8, 9]. Therefore, efforts should be made to identify strategies to manage TT levels.

Given that T is a cholesterol-derived steroid hormone from Leydig cells and adrenals [10], it is possible to speculate that both cholesterol intake and serum total cholesterol (TC) may be associated with TT levels. However, to date, no study has evaluated these associations in a representative sample. The limited evidence as to whether cholesterol intake has an effect on TT levels has been demonstrated by a few randomized clinical trials [11, 12]. These studies showed that the increment of egg intake [11] or ketogenic diet intervention [12], which are nutritional approaches that raise cholesterol intake, increased TT levels in trained (resistance training) young men [11, 12]. However, although randomized clinical trials have an important scientific evidence level, it is not yet possible to conclude whether these increases in TT levels occurred due to an isolated effect of cholesterol intake or because other nutrients that can also have effects on TT levels were also more ingested, such as fats [13–15]. For example, Wilson et al. [12] demonstrated that a ketogenic dietary intervention, which increased total fat intake and (likely) cholesterol intake, increased TT levels. However, since this study did not evaluate cholesterol intake, it is not possible to conclude whether T levels increased by a higher intake of fat, cholesterol, or both. In addition, a recent study showed that increased intake of cholesterol through daily intake of 3 whole eggs (672 mg cholesterol from eggs, but ~ 842 mg/day from whole diet) for a 12-week increased TT levels. However, considering that this elevated intake of cholesterol is not usually consumed at the population level [16], it is not known whether habitual intake of cholesterol is associated with TT levels. Furthermore, the aforementioned studies [11, 12] have a small sample size (*n* ≤ 30) and do not provide evidence for the untrained (resistance training) population.

Therefore, studies evaluating the association between habitual cholesterol intake and TT levels in a representative sample adjusting for important confounders may contribute to the understanding of this issue at the population level. Thus, the present study aimed to evaluate whether cholesterol intake and TC levels (which are partially predicted by cholesterol intake) are associated with serum TT levels in a representative sample of US men from the National Health and Nutrition Examination Survey (NHANES) 2013–2014.

## Methods

### Survey and participants

**Fig. 1:**
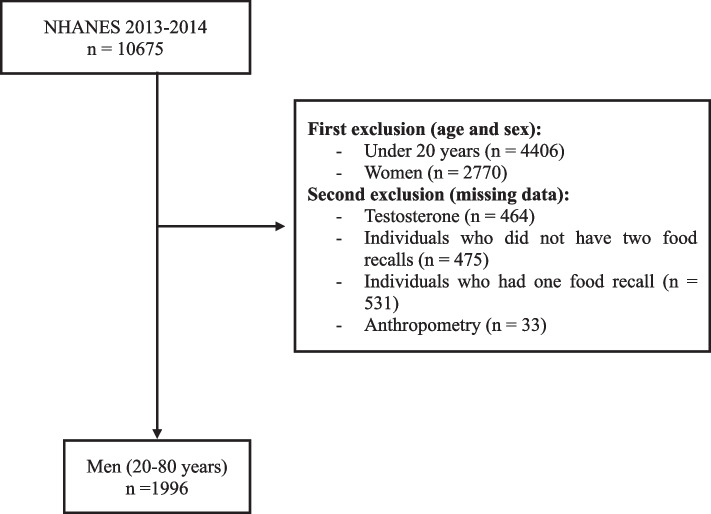
Flowchart of the sample selection from NHANES 2013–2014

Data were obtained from the National Health and Nutrition Examination Survey (NHANES) 2013–2014. NHANES is a cross-sectional survey conducted by the National Center for Health Statistics of Centers for Disease Control and Prevention based on a multistage, complex pooled, stratified probability sampling design to select a representative sample of civilians from non-institutionalized U.S. Initially, participants conducted interviews at home, which were administered by trained staff, and then all participants attended a mobile testing clinic, where trained staff collected anthropometric and biochemical data [17]. A total of 10,675 individuals were evaluated in NHANES 2013–2014. For the present study, women, individuals under 20 years old, individuals with missing data for TT levels, individuals who did not have two dietary recalls or who had one dietary recall, and individuals who did not have anthropometric measures were excluded. Thus, 1996 men aged 20 to 80 years were evaluated in the present study (Fig. 1). NHANES is a public dataset, and all participants provided written informed consent, consistent with approval from the National Center for Health Statistics Research Ethics Review Board (protocol #2011–17 for NHANES 2013–14).

### Anthropometrics

The body weight and height of each participant were obtained according to Lohman’s protocol to calculate the body mass index (BMI) [18].

### Total testosterone and serum cholesterol

TT values were selected for men who completed the morning laboratory test to better represent TT levels due to the diurnal variation of these hormones. TT levels were measured by the liquid chromatography method coupled with tandem mass spectrometry [19]. Serum TC levels were analyzed in venous samples collected following a standardized protocol, which were measured using coupled enzymatic reactions [20].

### Dietary intake

Food intake was obtained for all individuals through two 24-h dietary recalls [21], in which the first dietary interview was conducted in person at the mobile testing clinic and the second one was collected by phone 3 to 10 days later using the automated multiple pass method [22]. This method consists of an interview in 5 steps, which includes quick list, forgotten foods, time and occasion, detail cycle and final probe. Additionally, USDA's Food and Nutrient Database for Dietary Studies (FNDDS) 2013–2014 was used for processing the 2013–2014 intakes. The FNDDS includes comprehensive information that can be used to code individual foods/beverages and portion sizes reported by participants and includes nutrient values for calculating nutrient intakes. The basis for the nutrient values in FNDDS are detailed in the documentation for FNDDS 2013–2014 available at http://www.ars.usda.gov/nea/bhnrc/fsrg. The National Cancer Institute (NCI) method [23] was applied to estimate the usual dietary intake. The NCI method provides acceptable estimates of the usual intake distribution using two 24-h recalls and is a more reliable form to evaluate the usual intake compared with 2-day mean values since the NCI method corrects for intraindividual variability [24]. We evaluated the intake of energy (kcal/day), carbohydrates (g/day), total protein (g/day), protein (g/kg/day), lipids (g/day), total fatty acids (g/day) and their main types (saturated, monounsaturated, and polyunsaturated fatty acids), cholesterol (mg/day), total omega-3 (g/day), linoleic acid (g/day), fiber (g/day), zinc (mg/day), vitamins D and A (mcg/day), and alcohol (g/day).

### Covariates of interest

We considered demographic characteristics such as age (years), race/ethnicity (non-Hispanic white or other), marital status (single/divorced/widowed/never married or married/living as married), education level (lower/higher secondary education and some college or more) and annual household income (0 to $19.999, from $20.000 to 54.999, from $55.000 to 74.999 or above $75.000) as confounders. In addition, morbidities and lifestyle conditions included in the present study were hypertension (yes or no), diabetes (prediabetes, yes or no), and smoking (yes or no). We evaluated the use of cholesterol-lowering drugs through a questionnaire. The individuals were asked if they were taking prescribed medicine at the moment of the interview, and then they were classified as “yes” or “no”. Physical activity was evaluated as vigorous or moderate using a questionnaire. Vigorous-intensity activity was considered when performing activities that caused large increases in breathing or heart rate, such as carrying or lifting heavy loads, digging or construction work, for at least 10 min continuously. Moderate activity was considered when performing activities such as moderate-intensity sports, fitness, or recreational activities that cause a small increase in breathing or heart rate, such as brisk walking, bicycling, swimming, or volleyball, for at least 10 min continuously. The physical activity variable was evaluated as “yes” or “no”. If the individuals did not perform any type of physical activity, that is, answered no to vigorous or moderate physical activity, they were classified as “no”. The intake of energy (kcal/day), total fat (g/d) and alcohol (g/day) were also considered confounders.

### Statistical analyses

Linear regression was used to compare demographic, health and behavior conditions, anthropometric and biochemical parameters, and food intake according to quintiles of cholesterol intake. Continuous variables are described as the mean and standard deviation, and categorical variables are described as the percentage and confidence interval. Linear regression models were used to estimate coefficients and 95% confidence intervals (95% CI) for TT by quintiles of dietary cholesterol intake or blood cholesterol levels. Linear regression analyses were also performed to evaluate the association of TT levels with continuous values of dietary cholesterol and blood cholesterol levels. Analyses were performed without (Model 1) and with adjustments for confounders (Model 2). Variables included as adjustments were age, BMI, race/ethnicity, educational level, marital status, annual household income, diabetes, hypertension, use of cholesterol-lowering drugs, physical activity, smoking, energy intake (kcal/day), total fat (g/day), and alcohol (g/day). A missing variable was created for annual family income, educational level, and smoking. Stata 14.0 software (StataCorp, College Station, TX, US) was used for statistical analysis, and a *P* value < 0.05 was considered significant. All statistical analyses were performed considering the weight of the examination sample [17].

## Results

### Subjects’ characteristics

Table 1 shows the characteristics of the participants according to the quintile of cholesterol intake (mg/day). Men ingesting more cholesterol were predominantly younger, but a smaller proportion of non-Hispanic white individuals consumed more cholesterol. In addition, a lower prevalence of hypertension and diabetes was observed in the highest quintiles of cholesterol intake. Individuals ingesting more cholesterol had higher body weight and BMI and ingested more energy, carbohydrates (g/day), protein (g/day and g/kg/day), total, saturated, monounsaturated, and polyunsaturated fats (g/day), total omega-3 (g/day), linoleic acid (g/day), zinc (mg/day) and vitamin A (mcg/day). For biochemical parameters, no differences were found for TC (mg/dL) and TT (ng/dL) values according to the quintile of cholesterol intake (mg).

**Table 1:** Sociodemographic, health conditions and habits, physical activity, anthropometric and body composition, and dietary intake of men by quintiles of dietary cholesterol (NHANES, 2013–2014)

**Variables**		**Dietary Cholesterol quintiles**					
	**Total**	**Q1**	**Q2**	**Q3**	**Q4**	**Q5**	***p-trend***
Dietary Cholesterol (mg)	346.2 (83.1)	238.1 (33.2)	296.1 (11.4)	333.9 (10.5)	377.9 (16.2)	473.8 (63.9)	** < 0.001**
Age, years	46.9 (16.88)	52.3 (19.08)	47.4 (17.25)	47.0 (16.78)	46.2 (14.57)	42.4 (15.65)	** < 0.001**
Non-Hispanic white, %	66.6 (60.6 – 72.1)	68.8 (62.3 – 74.4)	71.6 (64.0 – 78.2)	67.1 (59.1 – 74.2)	67.5 (60.6 – 73.7)	57.9 (48.6 – 66.6)	**0.013**
***Marital status, %***							0.276
Single/divorced/widowed/never married	32.8 (28.7 – 37.1)	31.2 (25.6 – 38.1)	32.3 (23.8 – 42.1)	33.1 (26.6 – 40.7)	28.9 (22.5 – 36.2)	38.4 (30.7 – 46.8)	
Married/living as married	67.1 (62.8 – 71.2)	68.7 (61.8 – 74.9)	67.6 (57.8 – 76.1)	66.6 (59.2 – 73.3)	71.0 – 63.7 – 77.4)	61.5 – 53.1 – 69.2)	
***Annual family income, %***							0.767
$0–19.999	14.2 (10.8 – 18.4)	16.7 (12.6 – 21.8)	16.1 (9.8 – 25.2)	9.4 (6.4 – 13.7)	12.2 (8.1 – 17.9)	17.6 (11.3 – 26.3)	
$20.000–54.999	33.7 (29.6 – 38.0)	38.9 (28.1 – 50.9)	28.2 (22.9 – 34.31)	41.5 (35.7 – 47.5)	28.0 (22.1 – 34.8)	32.5 (27.3 – 38.1)	
$55.000–74.999	11.3 (8.8 – 14.3	9.9 (5.5 – 17.2)	12.5 (6.6 – 22.4)	13.8 (8.1 – 22.7)	9.5 (62.05 – 14.4)	10.2 (6.4 – 15.9)	
Over $75.000	37.5 (31.5 – 44.0)	31.4 (25.7 – 37.8)	40.2 (32.5 – 48.4)	32.9 (27.3 – 39.0)	47.4 (38.0 – 57.0)	34.1 (24.0 – 45.9)	
Missing	3.1 (2.1 – 46.64)	2.8 (0.9 – 8.1)	2.8 (1.0 – 7.4)	2.1 (0.9 – 4.6)	2.6 (1.4 – 4.8)	5.4 (3.1 – 9.1)	
***Educational level, %***							0.348
High school graduate or under	35.8 (30.8 – 41.1)	39.5 (30.7 – 49.1)	34.6 (25.4 – 45.3)	38.8 (31.5 – 46.5)	27.5 (20.9 – 35.2)	39.6 (32.3 – 47.4)	
Some college or above	64.1 (58.8 – 69.1)	60.4 (50.8 – 69.2)	65.3 (54.6 – 74.5)	61.1 (53.3 – 68.3)	72.4 (64.7 -79.0)	60.3 (52.5 – 67.6)	
Missing	0.005 (0.004 – 0.05)	-	-	0.002 (0.0003 – 0.02)	-	-	
***Health conditions and habits***							** < 0.001**
Hypertension, %	34.3 (30.5 – 38.4)	40.1 (32.8 – 47.8)	36.8 (29.8 – 44.4)	34.0 (27.8 – 40.9)	34.9 (27.3 – 43.4)	26.3 (20.7 – 32.8)	
Diabetes, %							**0.040**
Prediabetes	3.1 (2.1 – 4.4)	12.6 (5.5 – 28.5)	6.2 (2.7 – 13.3)	16.0 (7.8 – 32.3)	35.1 (15.3 – 78.2)	27.2 (12.2 – 59.3)	
Yes	9.1 (7.3 – 11.4)	13.2 (9.3 – 18.3)	9.9 (6.4 – 15.0)	8.8 (4.5 – 16.7)	5.8 (3.9 – 8.6)	8.7 (6.0 – 12.5)	
No	87.7 (85.2 – 89.8)	85.5 (80.8 – 89.2)	83.8 (75.3 89.7)	89.5 (81.7 – 94.1)	90.6 (86.0 – 93.7)	88.4 (84.0 – 91.8)	
Smoking %							0.707
Yes	19.0 (16.3 – 22.0)	21.4 (13.2 – 32.9)	18.7 (14.2 – 24.1)	16.4 (13.0 – 20.6)	21.2 (15.9 – 27.6)	17.6 (11.9 – 25.4)	
Missing	0.009 (0.001 – 0.08)	-	0.004 (0.0005 – 0.03)	-	-	-	
***Physical activity %***							0.148
Yes	55.9 (52.4 – 59.3)	49.5 (43.3 – 55.7)	56.4 (48.8 – 63.6)	53.6 (45.4 – 61.6)	61.4 (51.8 – 70.1)	57.4 (48.9 – 65.5)	
No	44.0 (40.6 – 47.5)	50.4 (44.2 – 56.6)	43.5 (36.3 – 51.1)	46.3 (38.3 – 54.5)	38.5 (29.8 – 48.1)	42.5 (34.4 – 51.0)	
***Anthropometrics***							
Weight, kg	89.2 (20.2)	86.1 (21.9)	87.7 (19.8)	91.5 (18.5)	89.0 (17.7)	91.2 (23.3)	**0.030**
Height, m	1.75 (0.7)	1.73 (0.7)	1.76 (0.7)	1.76 (0.6)	1.75 (0.7)	1.76 (0.7)	0.106
Body mass index, kg/m^2^	28.8 (6.0)	28.4 (6.8)	28.2 (6.4)	29.4 (5.2)	28.9 (5.0)	29.2 (7.0)	**0.046**
***Dietary intake***							
Energy, kcal/day	2399.6 (516.4)	2006.9 (453.4)	2264.4 (399.4)	2360.0 (353. 6)	2531.4 (430.2)	2789.1 (605.1)	** < 0.001**
Carbohydrate, g/day	279.8 (69.7)	251.6 (67.3)	269.3 (56.3)	279.9 (54.9)	288.0 (66.4)	306.9 (89.9)	** < 0.001**
Protein, g/day	96.4 (20.3)	76.2 (14.5)	89.9 (13.9)	94.2 (14.2)	103.2 (14.4)	116.1 (21.1)	** < 0.001**
Protein, g/kg	1.13 (0.3)	0.9 (0.2)	1.1 (0.3)	1.1 (0.2)	1.2 (0.3)	1.3 (0.4)	** < 0.001**
Lipids, g/day	92.0 (20.7)	74.5 (17.5)	85.3 (14.7)	89.4 (14.5)	99.1 (16.9)	109.5 (22.3)	** < 0.001**
Saturated fat, g/day	29.8 (7.2)	23.6 (5.4)	27.5 (4.9)	29.1 (5.9)	32.4 (6.1)	35.7 (8.3)	** < 0.001**
Monounsaturated fat, g/day	32.3 (7.3)	26.5 (6.7)	30.1 (5.6)	31.4 (5.3)	34.6 (5.7)	38.3 (7.7)	** < 0.001**
Polyunsaturated fat, g/day	21.1 (4.7	17.9 (4.5)	19.9 (3.8)	20.3 (3.9)	22.3 (4.2)	24.3 (4.8)	** < 0.001**
Total ω-3, g/day	2.3 (0.4)	1.9 (0.4)	2.1 (0.3)	2.2 (0.4)	2.4 (0.4)	2.6 (0.4)	** < 0.001**
EPA, g/day	0.026 (0.004)	0.023 (0.004)	0.025 (0.004)	0.026 (0.004)	0.026(0.004)	0.028 (0.005)	** < 0.001**
DHA, g/day	0.078 (0.026)	0.061 (0.018)	0.701 (0.024)	0.076 (0.024)	0.081 (0.023)	0.099 (0.02)	** < 0.001**
ALA, g/day	1.9 (0.4)	1.6 (0.4)	1.8 (0.3)	1.9 (0.3)	2.0 (0.4)	2.2 (0.4)	** < 0.001**
Linoleic acid, g/day	18.5 (4.3)	15.8 (4.1)	16.6 (3.6)	17.9 (3.6)	19.7 (3.8)	21.3 (4.3)	** < 0.001**
Fiber, g/day	18.7 (6.0)	17.8 (7.2)	18.2 (5.4)	18.7 (5.7)	18.8 (5.1)	20.2 (6.7)	0.014
Zinc, mg/day	12.9 (2.9)	10.8 (2.9)	12.1 (2.2)	12.6 (2.1)	13.6 (2.6)	15.0 (3.3)	** < 0.001**
Vitamin D, mcg/day	25.5 (35.6)	24.4 (36.6)	20.4 (19.4)	34.6 (55.8)	23.8 (28.9)	25.1 (22.5)	0.678
Vitamin A, mcg/day	693.8 (613.0)	537.2 (681.5)	638.9 (516.8)	596.3 (457.1)	727.3 (505.1)	963.6 (815.9)	** < 0.001**
Alcohol, g/day	12.7 (15.7)	10.6 (1.0)	13.3 (1.5)	12.2 (0.9)	12.4 (1.0)	14.4 (1.4)	**0.134**
***Biochemical parameters***							
Total Cholesterol, mg/dL	187.2 (39.9)	180.5 (44.0)	183.3 (40.7)	191.4 (33.7)	193 (41.0)	185.9 (39.3)	0.055
Total Testosterone, ng.dL	412.9 (176.9)	400.4 (167)	413.2 (177)	402.7 (160)	427.8 (200.2)	418.3 (166.1)	0.158

*ALA* Alpha linolenic acid, *AMMI* Appendicular skeletal muscle mass index, *DHA* Docosahexaenoic acid, *EPA* Eicosapentaenoic acid, *LDL* Low-density lipoprotein, *SHBG* Sex hormone binding globulin. Data described as the mean (standard deviation) or percentage (confidence interval)

### Cholesterol intake and total testosterone levels

**Fig. 2:**
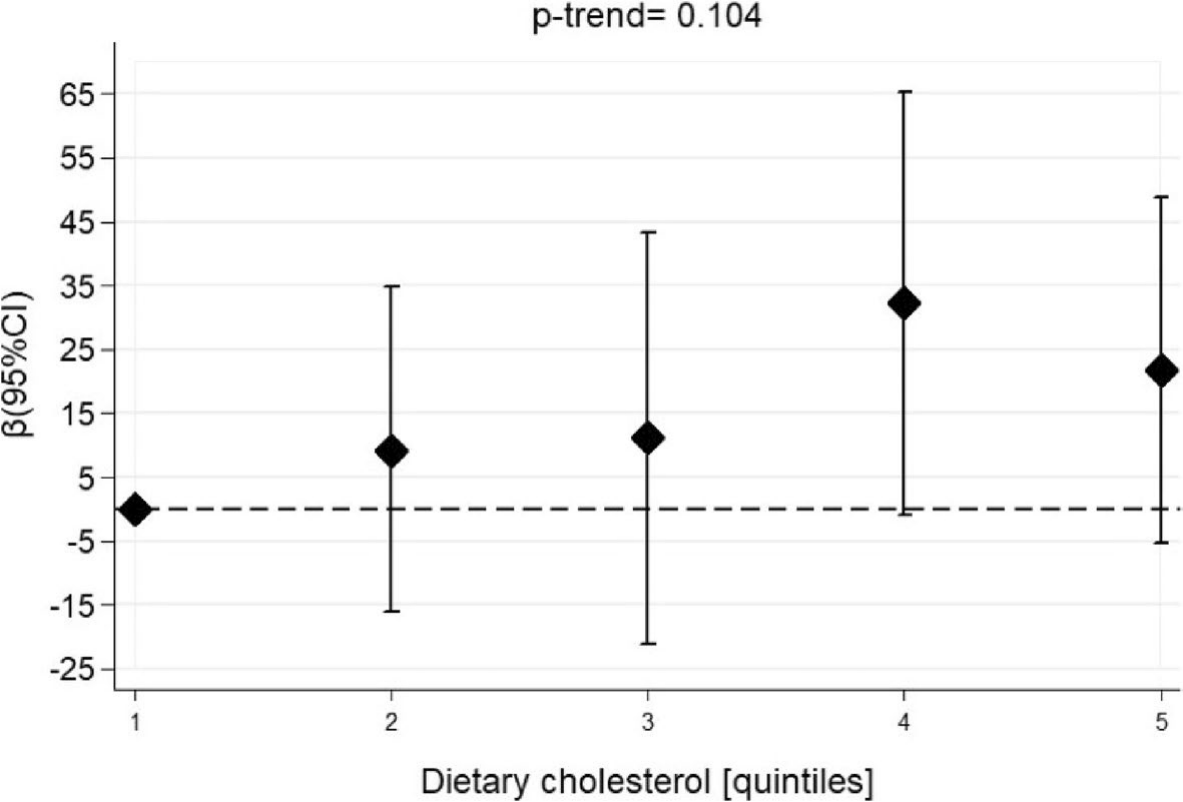
Linear regression between quintiles of dietary cholesterol and total testosterone levels. NHANES, 2013–2014. Adjusted for energy (kcal/d), total fat (g/day) and alcohol intake, smoking, age, physical activity, family income, marital status, race, educational level, diabetes, hypertension, BMI, and use of cholesterol-lowering drugs. Quintiles of dietary cholesterol (mg): quintile 1: 89.4 – 273.6; quintile 2: 273.7– 314.6; quintile 3: 314.7– 353.0; quintile 4: 353.1 – 411.1 and quintile 5: 411.4 – 835.2

No associations were observed between the quintile of cholesterol intake and TT in the unadjusted analyses (Supplementary Table 1) or when adjusted for confounders (Fig. 2 and Supplementary Table 1). Continuous values of cholesterol intake were also not associated with TT levels after adjustments for confounders (Table 2).

**Table 2:** Linear regression of continuous values of dietary and blood cholesterol with total testosterone levels in men aged 20–80 y. NHANES, 2013–2014

	**Model 1**			**Model 2**		
	β	95%CI	*p* value	β	95%CI	*p* value
Dietary cholesterol (mg/day)	0.081	-0.022; 0.184	0.115	0.089	-0.233; 0.200	0.112
Blood cholesterol (mg/dL)	-0.150	-0.503; 0.203	0.379	-0.005	-0.298; 0.287	0.968

*Model 1:* Without adjustment. *Model 2:* adjusted for energy (kcal/d), total fat (g/d) and alcohol intake, smoking, age, physical activity, family income, marital status, race, educational level, diabetes, hypertension, BMI, and use of cholesterol lowering drugs. Values are shown as coefficients confidence intervals (95% CI)

### Serum cholesterol and total testosterone levels

**Fig. 3:**
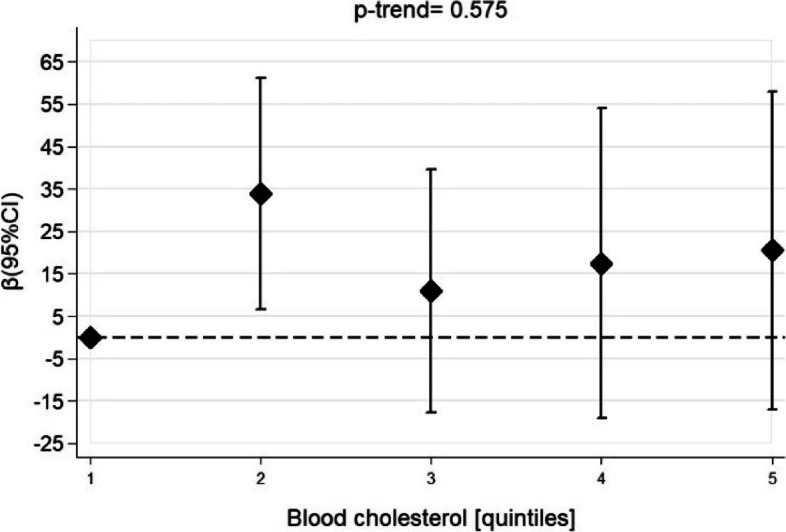
Linear regression between quintiles of blood cholesterol and total testosterone levels. NHANES, 2013–2014. Adjusted for energy (kcal/d), total fat (g/day) and alcohol intake, smoking, age, physical activity, family income, marital status, race, educational level, diabetes, hypertension, BMI, and use of cholesterol-lowering drugs. Quintiles of blood cholesterol (mg/dL): quintile 1: 82 – 150; quintile 2: 151– 173; quintile 3: 174 – 194; quintile 4: 195 – 218 and quintile 5: 219 – 362

Quintiles of TC were not associated with TT levels in the unadjusted analyses (Supplementary Table 1) or when adjusted for confounders (Fig. 3 and Supplementary Table 1). Continuous values of TC levels were also not associated with TT levels after adjustments for confounders (Table 2).

## Discussion

The main result of the present study was that cholesterol intake and TC levels were not associated with serum TT levels in men (20 to 80 years old) from a representative US sample. The lack of association was observed even considering an average cholesterol intake (347 mg/day) above that recommended by some guidelines, such as the Dietary Guidelines for Americans, which recommended not exceeding 300 mg/day of dietary cholesterol intake [25]. However, there is no current limit on cholesterol intake, as the 2015–2020 Dietary Guidelines for Americans removed the recommendation to restrict cholesterol intake to up to 300 mg/day [26]. Although the mean values of cholesterol intake from the overall sample size (347 mg/day) and the highest quintile (474 mg/day) exceeded the previous cholesterol intake threshold by 16 to 58%, respectively, we did not find a positive association with serum TT levels. Our results therefore do not support the hypothesis that a higher cholesterol intake is associated with higher TT levels, although cholesterol is the primary precursor for T synthesis [27]. In addition, we also did not observe an association between serum cholesterol (that is partially predicted by dietary cholesterol) and TT levels, which shows that TT does not seem to be associated with cholesterol, independent of the form that it was evaluated.

To the best of our knowledge, the present study is the first to evaluate the cross-sectional association between cholesterol intake and TT levels in a representative US sample. This limits direct comparison with studies that have the same design (cross-sectional), but other studies have evaluated a possible effect of cholesterol intake on TT levels. A prospective study showed that reducing cholesterol intake from ~ 633 mg/day to ~ 342 mg/day for 6 weeks was able to decrease serum TT from ~ 655 ng/dL to ~ 557 ng/dL in healthy middle-aged men (40 to 49 years old) [28]. However, this decrease in serum TT levels is within the range considered normal for men (~ 300 to 800 ng/dL) and likely has no clinical relevance [29].

Considering that eggs are one of the main food sources of dietary cholesterol, hypothetically, the intake of eggs itself could increase TT levels [30]. A recent study evaluating physically active eugonadal young men showed that daily intake of 3 whole eggs (672 mg cholesterol from eggs, ~ 842 mg/day from whole diet) for a 12-week strength-training program increased postworkout serum TT levels (~ 240 ng/dL) compared to an ~ 70 ng/dL increase for the control group (6 egg whites; 0 mg cholesterol from eggs, ~ 285 mg/day from whole diet) [11]. Although this [11] and the present study do not have the same study design, which limits comparisons of the results, it is possible to observe that a high intake of cholesterol per day (~ 842 mg/day) seems to be necessary to promote an increase in TT levels [11]. In the present study, the lack of association can partially be explained by a lower intake of cholesterol observed in the last quintile (mean intake of 474 mg/day). Therefore, our study shows that habitual dietary cholesterol intake is not associated with TT levels, but based on other studies, it is possible to speculate that a greater intake of cholesterol could promote increases in TT levels. However, it is unclear whether these increases in TT levels have important clinical implications [30].

Interventional studies using other dietary models with higher cholesterol intake should also be considered. The ketogenic diet is the dietary model with the highest cholesterol intake [31]. Overall, 500 to 1000 mg of cholesterol per day is expected in the ketogenic diet, but the common ketogenic diet is a cluster of high intake of cholesterol, total fat, and saturated fatty acids [12, 31], which limits the extrapolation of the isolated effect of cholesterol intake on TT levels. Wilson et al. demonstrated that a ketogenic dietary intervention (~ 220 g/day of total fat; ~ 110 g/day of saturated fatty acids) associated with 12 weeks of strength training in trained young men increased TT levels by ~ 120 ng/dL compared to subjects who underwent a low-fat Western diet (~ 84 g/day of total fat; ~ 37 g/day of saturated fatty acids) [12]. Conversely, these authors did not show the data for cholesterol intake, although a high intake could be conceivable by virtue of the high intake of total and saturated fatty acids in the ketogenic diet.

Given the relationship between cholesterol intake and total fat, a critical appraisal of total fat consumption and its effects on TT levels is of great interest. A study showed that reducing total fat intake from ~ 112 to ~ 40 g/day promoted a decrease in serum TT concentrations from ~ 438 to ~ 386 ng/dL in healthy middle-aged men (50 to 63 years old) [13]. This result is in line with a recent meta-analysis of intervention studies that confirmed the inverse relationship between low-fat diets and TT levels in men [14]. In terms of an epidemiological basis, while our study did not show a relationship between cholesterol intake and TT levels, a recent study (*n* = 3,128) employing NHANES analyses (1999 to 2000, 2003 to 2004, and 2011 to 2012) observed that men (*n* = 457) following a low-fat pattern had lower TT levels than men in general (410.8 ± 8.1 vs. 443.5 ± 7.3, *p* = 0.005), even when controlling for confounding variables (comorbidities, age, BMI, and activity level) [32]. In the present study, we observed that individuals ingesting more cholesterol also ingested more fat, which would be an important confounder. However, we showed that the lack of association between cholesterol intake and TT was independent of total fat intake.

### Study strengths and limitations

The present study has limitations. First, the study design is cross-sectional, and causality cannot be established. Second, we did not focus on patients with hypogonadism, and thus, we cannot state whether the increase in cholesterol intake is useful for males with low TT levels. As strengths, our study evaluated a representative sample of men from the USA. The current population of individuals represents a heterogeneous sample of the real practical scenario and hence depicts clinical applicability. The usual cholesterol intake was modeled through the NCI method based on data from two 24HRs, which reduces bias caused by measurement error in estimated associations between usual dietary intakes and health outcomes [23]. All analyses were adjusted for important confounders. We performed regression analyses according to quintiles of cholesterol and evaluated the continuous values. This shows that TT levels are not associated with dietary and blood cholesterol independent of the form in which the analyses were performed.

## Conclusion

Dietary cholesterol intake and TC levels were not associated with TT levels in men from the US. Therefore, although testosterone is partially synthesized from cholesterol, individuals with higher habitual cholesterol intake do not have higher testosterone levels. Longitudinal research is needed to evaluate whether an isolated increase in cholesterol intake can change TT levels in men.

### Supplementary Information

**Additional file 1:** **Supplemental Table 1.** Linear regression of quintiles of dietary cholesterol and serum total cholesterol levels with total testosterone levels in men aged 20-80 y from the NHANES, 2013-2014.

## Data Availability

The dataset generated and analyzed during the current study is available from the corresponding author upon reasonable request. In addition, NHANES is a public dataset that can be freely accessed at https://wwwn.cdc.gov/nchs/nhanes/continuousnhanes/default.aspx?BeginYear=2013.
