# Supplementary material for: Cholesterol intake and serum total cholesterol levels are not associated with total testosterone levels in men: a cross-sectional study from NHANES 2013–2014
Source: Lipids Health Dis. 2023 Oct 5;22:168. doi: 10.1186/s12944-023-01928-7 (PMC10552423; doi:10.1186/s12944-023-01928-7)
Supplement: Supplementary file 1 — Additional file 1: Supplemental Table 1. Linear regression of quintiles of dietary cholesterol and serum total cholesterol levels with total testosterone levels in men aged 20-80 y from the NHANES, 2013-2014. [file 12944_2023_1928_MOESM1_ESM.docx]

**Supplementary Table 1.** Linear regression of quintiles of dietary cholesterol and serum total cholesterol levels with total testosterone levels in men aged 20-80 y from the NHANES, 2013-2014.

|  |  | **Model 1** | | | | | **Model 2** | | | | | |
| --- | --- | --- | --- | --- | --- | --- | --- | --- | --- | --- | --- | --- |
|  | **Q1** | **Q2** | **Q3** | **Q4** | **Q5** | **p-trend** | **Q1** | **Q2** | **Q3** | **Q4** | **Q5** | **p-trend** |
| **Dietary cholesterol** | ref | 12.7  (-19.6; 45.1) | 2.3  (-25.7; 30.2) | 27.4  (-3.4; 58.7) | 17.9  (-11.1; 46.9) | 0.158 | ref | 9.3  (-16.1; 34.7) | 11.1  (-21.1; 43.4) | 32.2  (-0.9; 65.3) | 21.7  (-5.3; 48.8) | 0.104 |
|  |  |  |  |  |  |  |  |  |  |  |  |  |
|  |  |  |  |  |  |  |  |  |  |  |  |  |
| **Serum cholesterol** | Ref | 46.9  (22.5; 71.2) | 9.6  (-27.8; 47.0) | -0.13  (-35.1; 34.8) | 10.9  (-36.3; 58.1) | 0.602 | ref | 33.9  (6.6; 61.1) | 10.9  (-17.6; 39.6) | 17.4  (-19.0; 54.0) | 20.6  (-16.9; 58.0) | 0.575 |

**Notes:** Model 1: Without adjustment. Model 2: adjusted for energy (kcal/d), total fat (g/d) and alcohol intake, smoking, age, physical activity, family income, marital status, race, educational level, diabetes, hypertension, BMI, and use of cholesterol-lowering drugs. Values are shown as coefficients and 95% confidence intervals (95% CI).
